# Interaction of titanium, zirconia and lithium disilicate with peri-implant soft tissue: study protocol for a randomized controlled trial

**DOI:** 10.1186/s13063-015-0979-4

**Published:** 2015-10-15

**Authors:** Katharina Kuhn, Heike Rudolph, Michael Graf, Matthias Moldan, Shaoxia Zhou, Martin Udart, Andrea Böhmler, Ralph G. Luthardt

**Affiliations:** Department of Prosthetic Dentistry, Center of Dentistry, Ulm University, Ulm, Germany; Department of Clinical Chemistry, Ulm University, Ulm, Germany; Institut für Lasertechnologien in der Medizin und Messtechnik, Ulm University, Ulm, Germany

**Keywords:** Biomolecular, Peri-implant crevicular fluid, Immunohistochemical markers, Lithium disilicate

## Abstract

**Background:**

Against the background of increasing use of dental implants, and thus an increasing prevalence of implant-associated complications, a deeper understanding of the biomolecular mechanisms in the peri-implant tissue is needed. Peri-implant soft tissue is in direct contact with transmucosal dental implant abutments. The aim of this trial is to distinguish the biomolecular and histological interactions of various dental abutment materials with peri-implant soft tissue.

**Methods/Design:**

The study is designed as a prospective, randomized, investigator-initiated clinical pilot trial with blinded assessment. We will ultimately include 24 eligible patients who opt for implant treatment to replace a single missing posterior tooth. Three months after implantation (submerged procedure), the study begins with the second-stage surgery. Each of the 24 patients will be given three different transmucosal abutments (zirconia, lithium disilicate, titanium) consecutively. The sequence in which the three materials are used is randomized. Peri-implant crevicular fluid is sampled weekly around the respective abutment for biomolecular analyses. After one month of wearing time, the stamping press from the second-stage surgery is used to gain a narrow gingival ring biopsy around the abutment for immunohistochemical analyses. The next abutment is then inserted. The same procedure is used for all three abutments. After sampling is completed, the patients will receive a definitive crown. The primary outcome measure of the trial is biomolecular detection of specific markers in the peri-implant crevicular fluid: matrix metalloproteinase 8, interleukin- 1β, polymorphonuclear elastase, and myeloid-related protein MRP8/14 (calprotectin). Secondary outcome measures include immunohistochemical analyses and clinical parameters.

**Discussion:**

The study design will allow us to perform correlation analyses between the clinical indices with biomarkers’ expression in the interface of the transmucosal abutments and the peri-implant soft tissue. A deeper understanding of the three abutment materials’ interactions with peri-implant soft tissue will help us understand the formation mechanisms of implant-associated complications and then develop prevention strategies.

**Trial registration:**

The trial is registered at the German Clinical Trial Register and the International Clinical Trials Registry Platform by the WHO under DRKS00006555 (Registered on 27 October 2014).

## Background

The prevalence of biological implant-associated complications rises with the increasing use of dental implants. A common biological complication is peri-implant mucositis [1, 2], with a prevalence of 50 % of all dental implant sites [3]. Biological implant-associated complications usually begin in the peri-implant soft tissue. Both connective tissue and epithelium of the peri-implant soft tissue are in direct contact with transmucosal implant abutments. Thus, the interaction between the respective abutment materials with the peri-implant soft tissue may favor, counteract, or not influence at all the development of peri-implant mucositis or of unfavorable mucosal structures such as an apical shift of the barrier epithelium [4, 5]. The particular interaction between abutment materials and soft tissue is likely to be affected by the abutments’ properties, such as bacterial adhesion [6–8], surface condition [9–12] (e.g., free surface energy, roughness), and soft tissue integration ability [4, 5]. There is only low-level evidence available on the interaction between different abutment materials with peri-implant tissues [13, 14]. It has been suggested in histological studies that the abutment materials may have an influence on the stability of peri-implant tissues [4, 5, 15], although clinical studies that compared titanium to aluminum oxide abutments [16, 17] and titanium to gold-alloy abutments [18] found no significant differences in the clinical parameters. Titanium and zirconia are well-established as abutment materials. Lithium disilicate ceramic has recently been introduced as abutment material [19, 20]. Accordingly, there is a lack of studies on lithium disilicate compared with titanium and zirconia as dental implant abutment material.

The abutment’s interaction with peri-implant soft tissue can be analyzed by quantifying possible inflammation processes. The use of biomolecular analysis of the peri-implant crevicular fluid (PICF) has been established [21, 22]. For the PICF marker analyses the biomarkers matrix metalloproteinase 8 (MMP-8), interleukin-1β (IL-1ß) and polymorphonuclear elastase (PMN-elastase) proved to be reliable indicators of peri-implant inflammation [22–28]. They also play important roles in oral wound healing [29–32]. The biomarker myeloid-related protein MRP8/14 (calprotectin) has been used only rarely in PICF marker evaluations [33]. Its correlation with periodontitis has been proven by means of gingival crevicular fluid analyses [34–37]. Its release from monocytes is induced by IL-1ß [38].

Only one recently published study [39] compared the interaction of two abutment materials (titanium versus zirconia) with peri-implant tissue by means of PICF marker analyses. No such investigation is available on the biomolecular interaction of lithium disilicate ceramic. Overall, little evidence is available on the biomolecular interactions between different abutment materials and peri-implant soft tissue. The aim of this study is to analyze the interactions at the interface of peri-implant soft tissue and three dental abutment materials (zirconia, lithium disilicate, titanium). The hypothesis is that this interface differs biomolecularly and immunohistochemically depending on the specific abutment material used.

## Methods/Design

The study protocol is reported according to the Consolidated Standards of Reporting Trials (CONSORT) statement [40]. The study was designed in accordance with the following guidelines:

- World’s Medical Association’s Declaration of Helsinki
- Clinical investigation of medical devices for human subjects – Good Clinical Practice (ISO 14155:2011)
- Guidelines of Good Clinical Practice (2001/20/EC)

The Ethics Commission of Ulm University approved the study design on 30 July 2013 (processing number 68/13).

## Trial design

This clinical trial was designed as a prospective, randomized investigator-initiated pilot trial to be conducted in one center. In all, 24 patients are intended to participate in the study after giving informed consent. The study design was reevaluated after the first three patients had passed the endpoint of the biomolecular and histological sampling. Since the first three patients required no adjustments post-treatment, it is feasible that for the treatment of the remaining 21 patients no further adjustments will be required.

### Participants

Participants will be 24 patients with a single missing tooth in the posterior area (premolar or molar) with both adjacent teeth in situ and who choose to undergo implant replacement. Patients will be included in the study provided they:

- Are between 18 and 75 years of age
- Have an edentulous space at least 7 mm in width
- Have primary implantation or implantation after two-stage or one-stage augmentation (sinus lift or minor lateral augmentation)
- Have gingiva ≥ 3 mm in height
- Are in need of prosthetic treatment
- Are legally competent

Patients will be excluded from the study if:

- Extensive lateral one-stage augmentation is required (two-stage augmentation with bone block and one-stage sinus lift or minor lateral augmentation is possible)
- They are smokers
- Implant insertion using an implant template before the beginning of the study is not possible
- The edentulous space < 7 mm in width (conservation of papilla)
- The gingiva < 3 mm in height at the lowest point
- There is no consent given for study participation
- A chronic disease is present
- They are pregnant
- There is evidence of alcohol or drug abuse

### Settings and locations where the data will be collected

The study is taking place at the Department of Prosthetic Dentistry, Center of Dentistry, Ulm University. Screening began in August 2013. So far, the first eight patients have received a single-tooth implant. Five of the eight have already finished the study. All patients gave informed consent. The biomolecular analyses will take place in the Department of Clinical Chemistry, Ulm University. The immunohistochemical analyses will be carried out at the Institut für Lasertechnologien in der Medizin und Messtechnik, Ulm University. Completion of the study (last patient out) is planned for mid-2016.

### Interventions

All 24 patients will have received the three abutments consecutively, each for a 1-month wearing period. The abutments are as follows: zirconia ceramic (Z) (Zenostar MO; Wieland Dental GmbH, Pforzheim, Germany; lot S13270); lithium disilicate ceramic (L) (IPS e.max Press; Ivoclar Vivadent, Schaan, Liechtenstein; lot S44695); titanium (T) (Zenotec Ti; Wieland Dental GmbH, Pforzheim, Germany; lot 20130305 4012). The sequence of the three materials is randomly assigned. The abutments are manufactured by luting hollow Z-, L-, or T-cylinders on Variobases™ (Straumann, Basel, Switzerland; lot: HE661/HK699) by means of Multilink® Hybrid Abutment cement (Ivoclar Vivadent, Schaan, Liechtenstein; lot: T10017). The abutments’ surface roughness is adjusted by polishing them to ensure conformity. The results are checked by Ra measurements of each abutment (Ra < 0.1 μm).

The examinations and specific data collection are shown in Table 1. Screening is performed before implantation to check the inclusion/exclusion criteria. Implantation is performed according to the standardized implantation procedure in the Department of Prosthetic Dentistry making use of the Straumann®-guided surgery implantation system (Straumann, Basel, Switzerland) in combination with an implantation template after three-dimensional implant planning (CoDiagnostix; Dental Wings GmbH, Chemnitz, Germany). Bone-level Straumann implants (SLActive, RC) are submerged.

**Table 1:** Examinations and collected data

Examination type	Examination time	Collected data
Screening	Before implantation; every patient eligible for the study regardless of his/her participation in the trial	Checking of the inclusion/exclusion criteria
Implantation	Before inclusion in clinical trial	
Pretreatment examination	Before randomization	Clinical control of implant site; informed consent
Baseline: second-stage surgery and insertion of first abutment	90 ± 14 days after implantation	Tissue sampling (control punch biopsy)
		Photo documentation
		X-ray control
PICF sampling	7, 14, 21, and 28 (±1) days after insertion of the first, second, and third abutment	PICF sampling
		Plaque Index (PI)
		Gingival Index (GI)
		Probing depth (PD)
		Bleeding on probing (BOP)
Tissue sampling and insertion of second and third abutments	2–5 days after the last PICF sampling around the first, second, and third abutment	Tissue sampling (ring biopsy)

Informed consent is given during the healing period (90 ± 14 days). Inclusion in the study and randomization takes place prior to the second-stage surgery (baseline). For the second-stage surgery, a stamping press is used, with a punch biopsy serving as a histological control specimen. The first abutment (Z, L, or T) is inserted (20 Ncm) according to the randomization process. The occlusal screw hole is provisionally closed by means of a piece of dental foam and Luxatemp Inlay (DMG, Hamburg, Germany), which is light-cured. These materials are not in contact with the gingiva.

**Fig. 1:**
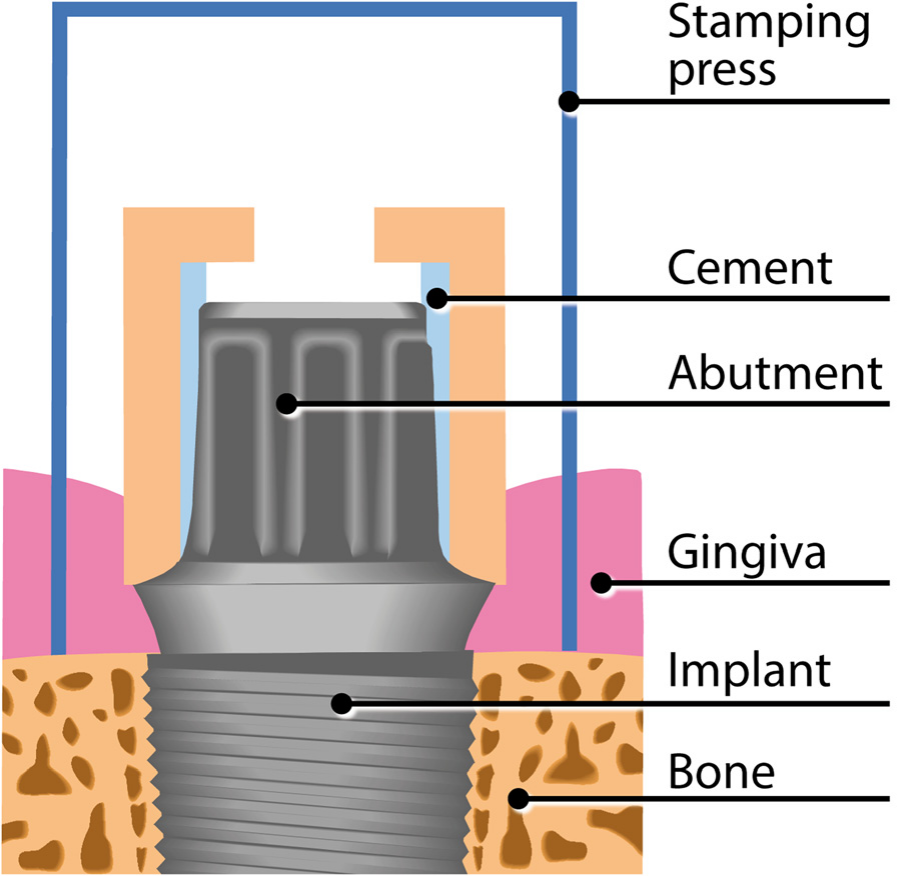
Sampling for the immunohistochemical analyses

**Fig. 2:**
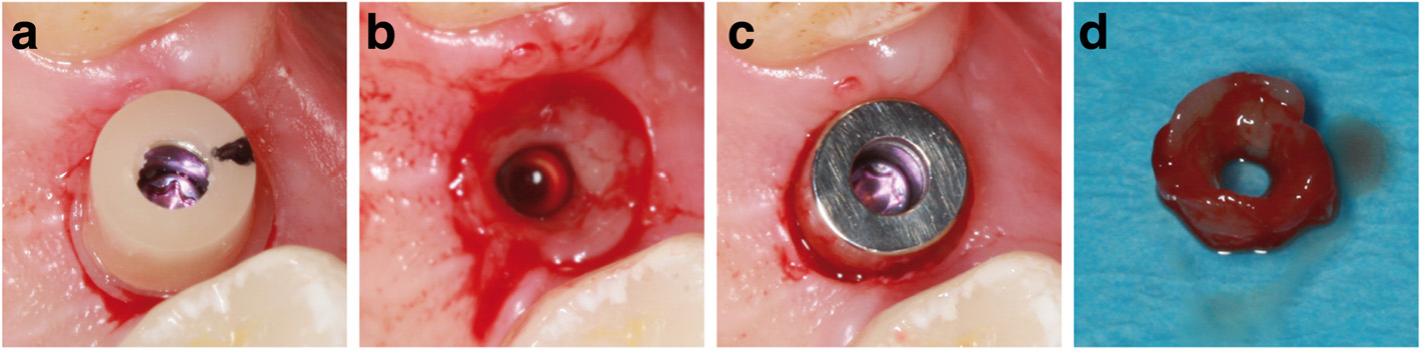
Tissue sampling. **a** Clinical situation after applying the stamping press around the zirconia abutment. **b** After removing the gingival ring biopsy and the zirconia abutment. **c** After inserting the titanium abutment. **d** Gingival ring biopsy

Peri-implant crevicular fluid (PICF) is sampled both buccally and lingually every week using paper strips (Periopaper; Oraflow Inc., New York, NY, USA). Before PICF sampling, supragingival plaque is carefully removed with cotton balls to eliminate the risk of plaque contamination. Cotton rolls are applied to isolate the sample from saliva. After air-drying the teeth, the paper strips are inserted into the sulcus/pocket until there is mild resistance and then left there for 30 seconds. Samples with visible blood contamination are discarded. In addition to weekly PICF sampling, clinical parameters are recorded for the implant and both adjacent teeth, including the Plaque Index (PI), Gingival Index (GI), probing depth (PD) using an electronic pressure-sensitive probe (FloridaProbe, Essen, Germany) at four sites (mesiobuccal, distobuccal, mesiolingual, distolingual), and bleeding on probing (BOP). After a 1-month wearing period, the abutment is exchanged for the next one. Before unscrewing the abutment, the stamping press from the second-stage surgery is used to gain a narrow (0.6 mm) gingival ring biopsy around the abutment for immunohistochemical analyses (Figs. 1 and 2). The same procedure as described above is repeated for the second and third abutments. After completion of the sampling for biomolecular and immunohistochemical analyses, the study is completed, and the patient receives a screw-retained lithium disilicate crown.

The three clinical investigators collecting the data received training before the first study visit to guarantee conformity.

#### Biomolecular analyses

The paper strips collected for biomolecular analyses are frozen immediately after sampling (−80 °C) and remain frozen until assayed. For the analyses, the PICF is released from the paper strip by means of analysis buffer. The incubation takes place on an orbital shaker on ice, and separation is performed by centrifugation. The supernatant is collected into a fresh tube. To exclude erythrocyte contamination, all extracts are examined with the Combur-Test® strip (Roche Diagnostics GmbH, Mannheim, Germany).

The PICF extracts are analyzed for biomolecular detection of specific markers: MMP-8, IL-1β, PMN-elastase and MRP8/14 (calprotectin). MMP-8 is analyzed with the Fluorokin MAP Multiplex Human MMP Panel (R & D Systems GmbH, Wiesbaden, Deutschland) in the Luminex 200 System (Bio-Rad Laboratories Inc., Hercules, CA, USA). The MMP-8 activity is detected by means of gelatine-zymography. IL-1ß is measured by the Bio-Plex Cytokine Assay Kit (Bio-Rad Laboratories Inc., Hercules, CA, USA) in combination with the Luminex 200 System. PMN-elastase is measured by means of the Human Elastase ELISA Kit (HyCult Biotechnology, Uden, Netherlands) and MRP8/14 is measured with the MRP8/14 ELISA Kit (Bühlmann Laboratories AG, Schönenbuch, Switzerland). The optical density for both kits is measured at 450 nm. The sensitivities are as follows: MMP-8, 8.9 pg/ml; IL-1β, 0.2 pg/ml; PMN-elastase, 0.4 ng/ml; MRP8/14, < 0.4 μg/ml.

#### Immunohistochemical analyses

Each of the four gingival biopsies per patient (one control punch biopsy and three ring biopsies around the abutment materials) (Fig. 2d) are equally divided into quarters for the immunohistochemical analyses. The biopsy specimens are fixed immediately in 4 % neutral buffered formalin at room temperature and left there for 24 hours before embedding them in paraffin. Three micrometer thick sections are prepared prior to deparaffinization. A citrate buffer (pH 6.1; Target Retrieval Solution, Dako, Carpinteria, CA, USA) is applied. A steamer is used for antigen retrieval (20 minutes) and the slides cool off for 20 minutes. A serum blocking solution (Histostain-Plus Bulk Kit, Invitrogen®, Camarillo, CA, USA; 20 minutes) is applied. The specimens are incubated with the primary antibodies Anti-Neutrophil Elastase (over night; 4 °C; 1:500), Anti-MMP 8 (over night; room temperature; 4 μg/ml), Anti-IL 1β (over night; room temperature; 1:12.5) or Anti-MRP8 antibody (over night; 4 °C; 1:750), respectively (Abcam®, Cambridge, Great Britain), targeting the same molecules which are analyzed by the biomolecular analysis. Hydrogen peroxide 0.3 % is applied to block endogenous peroxidase (10 minutes). A secondary biotinylated antibody (Histostain-Plus Bulk Kit, Invitrogen®, Camarillo, CA, USA) is used (10 minutes). Streptavidin conjugated with horseradish peroxidase (Histostain-Plus Bulk Kit, Invitrogen®, Camarillo, CA, USA) is applied (15 minutes). An AEC chromogen (AEC Single Solution, Invitrogen®, Camarillo, CA, USA) is applied (15 minutes) for final staining. The specimens are then counterstained with hematoxylin and mounted with Aquatex (Merck, Darmstadt, Germany). Negative controls are obtained by treating the sections similarly but, instead of the primary antibodies, Tris-buffered saline (TBS) is used. The staining is performed in serial sections from each specimen (e.g., lingual quarter of the control punch biopsy).

A digital image analysis system, which consists of a light microscope Axiophot (Carl Zeiss Jena, Oberkochen, Germany) and a digital color camera ProgRes C12 plus (Jenoptik, Jena, Germany) connected to a computer equipped with PeogRes-Software, is used to obtain images of the microscopic samples (magnification 100× to 400×). Morphometric analyses are performed using the software ImageJ (National Institutes of Health, Bethesda, MD, USA) with the plugins Grid and CellCounter as previously described [41].

### Outcomes

The primary outcome measure is the biomolecular detection of specific markers (MMP-8, IL-1β, PMN-elastase, MRP8/14) in the PICF samples. Secondary outcome measures include immunohistochemical analyses and clinical parameters (PI, GI, PD, BOP).

### Sample size

The study is biometrically categorized as a pilot study because of the sample size of 24 patients. During establishment of the study design, there were no clinical results on which a biometrical sample size estimation could be based. The sample size has, therefore, been set at 24 patients. The weekly PICF sampling both buccally and lingually results in 24 PICF samples from each patient. Four histological specimens (one control punch biopsy and three ring biopsies) are to be collected from each patient.

### Randomization

Randomization is performed with a threefold crossover design. The sequence of the three abutment materials is randomly assigned, resulting in six groups (ZLT, ZTL, LZT, LTZ, TZL, TLZ) with four samples for each. The study is stratified into the two gender groups.

Randomization concealment is ensured because a clinic staff member not involved in the clinical trial performs the allocation according to a randomization list.

Any eligible patient is recorded in a screening list maintained by the clinical investigators. Once a patient has given informed consent, a patient number and randomization are requested from the staff member performing the allocation according to the randomization list. The clinical investigator enters the patient number into the patient list, which also includes the appointments of each patient. The patient number and sequence of the abutment materials are transferred into the Case Report File of the baseline visit (second-stage surgery).

### Blinding (masking)

Masking is not possible for the dentist or the patient because of the visually distinguishable abutment materials. All analyses, however, are to be done masked because of the different personnel and spatial separation.

### Statistical methods

An explorative data analysis will be performed. The data will be analyzed descriptively by means of absolute and relative frequency and medians and means, respectively, as well as measures of dispersion. The analysis of the primary and secondary outcome measures will be performed using adequate statistical tests according to the distribution patterns. Power analyses and sample size estimation will be done for similar clinical studies in the future.

## Discussion

Against the background of a high prevalence of biological peri-implant complications [3], the mechanisms of the interface between peri-implant soft tissue and transmucosal abutments are of interest. The aim of the present clinical study is to obtain a deeper understanding of the interaction mechanisms via PICF marker analyses and immunohistochemical analyses.

To date, only one recently published cross-sectional clinical study [39] has investigated the interaction of abutment materials (titanium versus zirconia) with peri-implant tissue by means of PICF marker analyses. No significant differences were found between the biomarkers’ quantity (except for leptin). The present study has only the biomarker IL-1β in common with the other clinical study. Clinical parameters were not recorded (except for the presence or absence of plaque) at the time of PICF sampling. The authors recognize the benefits of a prospective, randomized clinical trial including the collection of clinical indexes for correlation with biomarker expression [39]. Our study design met those requirements. Moreover, we included a third material, lithium disilicate ceramic, which is not as well- established as titanium and zirconia for use as abutment material. It has been shown, however, to be an increasingly relevant material for abutments [19, 20]. Titanium serves as “gold standard.”

PICF marker analyses have been established to detect peri-implant inflammation [21, 22]. For the PICF marker analyses, we use three well-established biomarkers (MMP-8, IL-1ß, PMN-elastase) and MRP8/14. To date, MRP8/14 has been used only rarely in PICF marker evaluations [33]. The combination of well-established biomarkers with a rather new one allows us to evaluate its suitability for PICF marker analyses.

The role of plaque formation must be taken into account in terms of dental materials’ interaction with peri-implant soft tissue. Plaque is associated with peri-implant mucositis [42, 43] and is correlated with elevated inflammatory markers in PICF [28, 44]. In our study, we clinically determine the PI for correlation analyses with the number of biomarkers in PICF, which has not been sufficiently investigated because of the wide range of biomarkers. The formation of biofilm on dental materials has been especially well-examined for titanium and zirconia [6–9, 45]. The results are inconsistent, however, as zirconia shows less bacterial adhesion and colonization in some studies [7, 45, 46] and no differences in others [6, 8]. In the context of plaque formation, the role of surface roughness also must be discussed. Excessive surface roughness of dental materials favors biofilm formation [11, 12] with more complex microbiota [10]. A threshold for the Ra value of abutment surfaces has been found (0.2 μm), however, below which no further significant changes occur in regard to plaque accumulation [9]. The interaction between the three abutment materials with the peri-implant soft tissue should not be biased by different surface roughness in the present study. Therefore, we adjusted the Ra values of all abutments for uniformity, as indicated in previous studies [8, 45], and well below the above-mentioned threshold.

Wound healing also influences the results of PICF marker analyses [47]. This aspect will not bias our study as the study design guarantees analogue wound healing conditions for all abutment materials. Each abutment is inserted immediately after a circular gingival biopsy is performed with a stamping press. Thus, each abutment material is circularly in contact with open wound edges, and the 1-month PICF sampling takes place during the early wound healing period for each of the three abutments. This is a clinically relevant procedure especially for the “one abutment – one time” concept, where the definitive abutment is inserted directly after implantation (direct loading) or directly after second-stage surgery (delayed loading) without removing it again [48–50].

The original biopsy material (punch biopsy from second-stage surgery) serves as histological control specimen as it has not been in contact with an abutment material so far. It will also be stained immunohistochemically to detect the four biomarkers.

Washout periods between the different abutment materials may help to exclude an influence of the preceding abutment material on the tissues. Washout periods may be performed either by wearing a “neutral” abutment for a specific time between the tested abutments or by permitting tissues to heal without an abutment and punch biopsy again before inserting the next abutment. Following discussion with biometricians, the additional punch biopsies and time on top of an already prolonged procedure were deemed ethically unjustifiable. In addition, the newly formed cells and tissue in direct contact with each abutment are most likely to be affected and are circularly removed by the stamping press. Thus, we renounced washout periods in the trial. However, an influence of the preceding abutment material on the biomolecular and immunohistochemical findings of the next abutment material cannot be fully excluded. To the author’s knowledge, there is no study which has tested this aspect yet. If our biometricians detect clear evidence of previous abutment influence (worst-case scenario) only the first abutment material (*n* = 8 for each abutment material) shall be used for analysis.

The study combines biomolecular and immunohistochemical analyses – both aiming to detect the same biomarkers – and clinical analyses. This design allows a site-specific correlation analysis between those two techniques for biomarker detection as well as clinical indices to monitor gingival health.

This study is the first randomized clinical trial to combine biomolecular, immunohistochemical, and clinical analyses to determine the environment around transmucosal abutments during wound healing in humans. Two well-established abutment materials (titanium and zirconia) are compared with a more recently introduced abutment material (lithium disilicate). A deeper understanding of abutment materials’ interactions with peri-implant soft tissue will help us comprehend the formation mechanisms of implant-associated complications and to develop prevention strategies.

## Trial status

The screening and recruiting for the study began in August 2013 and is still ongoing. To date, the first eight patients have received a single-tooth implant. Five of the eight have finished the study.

## Abbreviations

BOP: bleeding on probing
CONSORT: Consolidated Standards of Reporting Trials
ELISA: enzyme-linked immunosorbent assay
GI: Gingival Index
IL-1β: interleukin-1β
L: lithium disilicate
MMP-8: matrix metalloproteinase 8
MRP: myeloid-related protein
PD: probing depth
PI: Plaque Index
PICF: peri-implant crevicular fluid
PMN-elastase: polymorphonuclear elastase
T: titanium
TBS: Tris-buffered saline
Z: zirconia
